# Management Considerations for Cervical Corpectomy: Updated Indications and Future Directions

**DOI:** 10.3390/life14060651

**Published:** 2024-05-21

**Authors:** Marco Foreman, Devon Foster, Wiley Gillam, Christopher Ciesla, Chris Lamprecht, Brandon Lucke-Wold

**Affiliations:** 1 Department of Neurosurgery, University of Florida, Gainesville, FL 32610, USA; marcoforeman@ufl.edu (M.F.); gillamw@ufl.edu (W.G.); chrislamprecht@ufl.edu (C.L.); 2 Herbert Wertheim College of Medicine, Florida International University, Miami, FL 33199, USA; dfost046@med.fiu.edu (D.F.); ccies003@med.fiu.edu (C.C.)

**Keywords:** cervical corpectomy, degenerative disc disease, anterior approach, skip corpectomy

## Abstract

Together, lower back and neck pain are among the leading causes of acquired disability worldwide and have experienced a marked increase over the past 25 years. Paralleled with the increasing aging population and the rise in chronic disease, this trend is only predicted to contribute to the growing global burden. In the context of cervical neck pain, this symptom is most often a manifestation of cervical degenerative disc disease (DDD). Traditionally, multilevel neck pain related to DDD that is recalcitrant to both physical and medical therapy can be treated with a procedure known as cervical corpectomy. Presently, there are many flavors of cervical corpectomy; however, the overarching goal is the removal of the pain-generating disc via the employment of the modern anterior approach. In this review, we will briefly detail the pathophysiological mechanism behind DDD, overview the development of the anterior approach, and discuss the current state of treatment options for said pathology. Furthermore, this review will also add to the current body of literature surrounding updated indications, surgical techniques, and patient outcomes related to cervical corpectomy. Finally, our discussion ends with highlighting the future direction of cervical corpectomy through the introduction of the “skip corpectomy” and distractable mesh cages.

## 1. Introduction

Cervical corpectomy is a surgical procedure involving nerve decompression that has been used for a variety of injuries related to the cervical spine. Common indications for cervical corpectomy include cervical degenerative disc disease (DDD), trauma, a tumor, infection, and the ossification of the posterior longitudinal ligament [1]. DDD is a medical umbrella term commonly used to describe osteoarthritis of the spine [2]. This review will focus on cervical DDD, which typically originates from cervical myelopathy or radiculopathy, and can be characterized by neck pain experienced in the upright position. Of note, cervical DDD is the primary cause of acquired disability in all adults above the age of 50 years old [3].

### Pathophysiology of Degenerative Disc Disease

**Figure 1:**
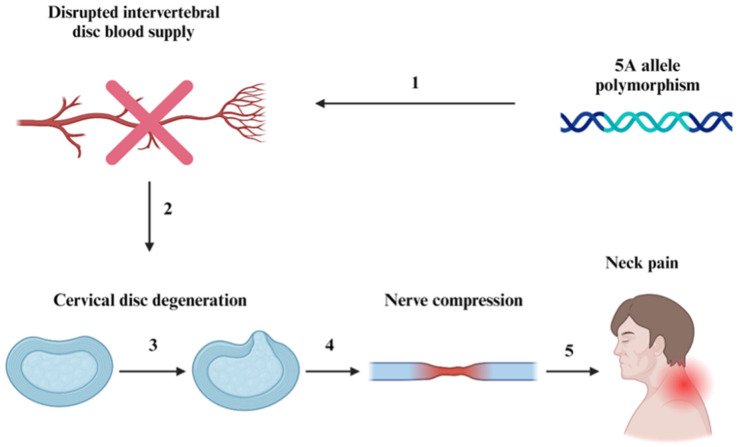
Pathophysiology of cervical degenerative disc disease. (1) and (2) display potential causative factors of cervical DDD. (3), (4), and (5) describe the consequences of cervical DDD, which ultimately lead to neck pain, which may radiate to other regions of the body (shoulders, arms, hands, etc.), causing numbness and tingling.

The primary injury resulting from cervical DDD requiring surgical intervention is cervical disc herniation, which typically begins with displacement of the nucleus pulposus at C5 or C6 [4]. Later, the degeneration of the annulus pulposus allows the nucleus pulposus to compress nearby nerves, leading to the destabilization of surrounding intervertebral discs [4,5]. According to some studies, genetic factors such as polymorphisms at the 5A allele may lead to increased risk of DDD [6]. In most cases, DDD results from a dysfunctional repair system due to a complication in the vascular pathway from the nucleus pulpous and inner annulus, as depicted in Figure 1 below.

## 2. History of Cervical Corpectomy

As the interventions for DDD continue to evolve, it is foundational to reflect on the history of cervical spine surgery, particularly that which led to the development of anterior cervical corpectomy.

### The Development of the Anterior Approach

The anterior approach to spinal surgery was not widely utilized until the 1950s due to the early popularity of the posterior approach [7,8]. However, as the knowledge of spinal pathophysiology, human biomechanics, and the widespread adoption of antibiotics emerged, surgical methodologies for DDD began to expand [8,9]. Throughout the 1950s, countless approaches were developed, including the pre-sternocleidomastoid approach, introduced in 1957, and the anterolateral approach in 1958 [8,10,11]. The most significant among these early procedures was the inaugural account of anterior cervical fusion, conducted by Robert Robinson and George Smith in 1955 to address symptoms of cervical spondylosis [7,8,10,12,13,14,15,16].

“The Smith–Robinson approach”, as it has been coined, was an anterior approach that proved to be superior to its predecessors [7,8,10,12,13,16]. Variations of this approach are still widely utilized in cervical corpectomies today, making it one of the most expansively utilized methods of gaining access to the cervical spine [13,16,17].

Over the past 30 years, there has been continual growth and adaptation in this approach. In 1959, Cloward further modified this technique to incorporate the use of a bone dowel to stabilize a newly designed interbody fusion technique [7,8,15,18]. Boni and Denaro built off Cloward’s technique to treat multilevel cervical pathologies. In 1969, they developed a multilevel corpectomy procedure involving an anterior decompression, corpectomy, and the insertion of an autologous graft to stabilize the vertebrae [8,15,18]. This procedure marked a significant step in the evolution of cervical corpectomies in modern medicine. The popularity of the Smith–Robinson approach has continued to grow through 1990–2000 with an increase of 17.8% to 69.5% of procedures shifting toward the anterior approach [18]. It has since elevated its status as the preferred approach (90% anterior approach and 10% posterior approach) of cervical spinal fusion from 2002 to 2009 [19,20]. This modern shift toward the anterior approach for cervical spinal surgery can largely be attributed to its increased safety and postoperative recovery, as well as the lower likelihood of injury to the spinal cord, facet joints, dura, and nerve roots compared to the posterior approach [21,22].

**Figure 2:**
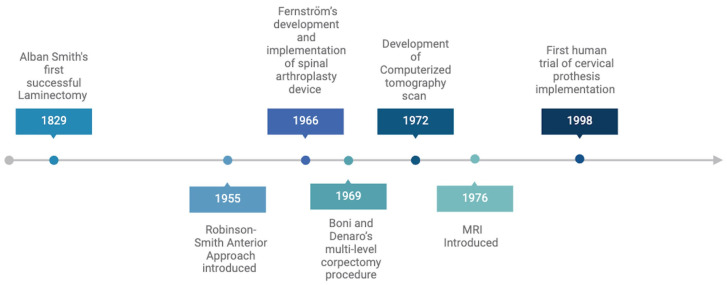
The evolution of cervical corpectomy. Outlined are key events in the modern development of spinal surgery techniques and technological advancements.

Moreover, the advent of advanced imaging modalities such as computed tomography and magnetic resonance imaging further propelled the field forward via the non-invasive visualization of key anatomical structures within the spine. Consequently, these developments unlocked new therapeutic interventions, such as the first human trial using a cervical prosthesis in 1998 by the Department of Medical Engineering at Frenchay Hospital in Bristol, United Kingdom [8,23]. A timeline describing the chronological progression of innovation and technological advancement in spinal surgery can be seen below in Figure 2.

## 3. Traditional Approaches to Degenerative Disc Disease

Given the storied history of spinal surgery and the recent understanding of the pathogenesis of DDD, several surgical techniques are observed in practice to address the pain and discomfort experienced by individuals afflicted with this pathology. Oftentimes, treatment is complex and requires a combination of several techniques. Some possible interventions include anterior cervical corpectomy and fusion (ACCF) with artificial disc replacement, ACCF with anterior cervical discectomy and fusion (ACDF), three-level discectomy, single corpectomy and discectomy, and two-level corpectomy [24,25]. Some studies have found cervical corpectomy to be most beneficial when combined with either single discectomy or single artificial disc replacement [24,25]. More broadly, another study reported that, from a sample of 185 patients, cervical corpectomy led to improvement in all cases of radiculopathy and most cases of myelopathy [26]. It is important to note that although studies have reported positive clinical outcomes (improved signs and symptoms; significant reduction in to complete loss of pain, motor deficits, and myelopathy; etc.) for post-cervical corpectomy patients, one meta-analysis found no significant difference in postoperative Japanese Orthopaedic Association (JOA) scores or the fusion rate between ACCF and ACDF [27]. The same study also found that ACDF may be associated with a decrease in both surgical- and instrument-related complication rates [27]. Further, others have found that ACDF is also associated with improved radiologic results [28,29].

While the aforementioned study and the like suggest that ACDF may be superior to ACCF, there is contradictory evidence that suggests the two procedures have similar outcomes. Particularly, two large systematic reviews by Jiang et al. and Wen et al. found ACCF to have both higher fusion rates and improved patient outcomes [30,31]. Clear clinical equipoise exists on which surgical treatment is superior and it is likely that ACCF and ACDF are each associated with their own strengths and weaknesses. Consequently, either may be appropriate depending on the etiology and severity of the pathology.

Adding to the complexity of the issue, cervical corpectomy is an ever-evolving surgical procedure. For example, anterior spinal column reconstruction following corpectomy used to be performed with autologous iliac crest bone. Recently, however, titanium mesh cages have become a more popular option for reconstruction [32]. In addition to changes in the procedure, cervical corpectomy has been utilized by surgeons for unique and novel indications. Of note, a five-level cervical corpectomy was conducted to correct severe kyphosis in a patient with neurofibromatosis [33]. This procedure was associated with no operative complications and the patient indicated considerable long-term improvements post-operation. Nonetheless, the primary indications for corpectomy have remained relatively consistent with disc degeneration, cervical spine trauma, and neoplastic diseases being the three most common [1,34].

Though cervical corpectomy has continued to be utilized for the same type of issues in the cervical spine, an appropriate application of the procedure requires a close examination of the patient’s condition. Some believe anterior cervical corpectomies to be the gold standard for larger lesions expanding outside of the disc space [35]. Conversely, smaller lesions within the boundaries of the disc space may require alternative techniques, such as anterior cervical discectomies. Notably, the anterior approach for both cervical corpectomies and discectomies has become critical for nearly all anterior cervical disc diseases. As such, its discovery in the 1950s revolutionized the field, greatly improving clinical utility and outcomes for corpectomies and discectomies alike [10,14,36,37]. In the proceeding section, updated indications for the employment of cervical corpectomy, the surgical technique, and patient outcomes will be explored.

## 4. Updated Indications, Surgical Technique, and Outcomes

### 4.1. Indications for Surgery

As previously discussed, cervical corpectomy is a procedure used to remove vertebral bodies and adjacent discs in the cervical spine, aiming to alleviate pressure on the spinal cord and nerve roots [1]. As such, cervical corpectomy has long served as a primary method in the treatment of upper spinal compression and traumatic injuries, as highlighted by Eleraky et al. [26]. Surgical intervention becomes necessary for patients who present with compression on nerve roots, the cervical cord, or both, with the intention of providing pain relief and alleviating neurological symptoms. This intervention is considered when patients have exhausted all non-operative treatment options.

In terms of imaging, surgical interventions for cervical corpectomies follow an MRI demonstrating severe spinal cord compression and myelopathy, CT scans revealing vertebral body and disc damage and structural abnormalities, X-rays indicating cervical spine instability or malalignment, or a CT myelogram demonstrating spinal cord or nerve root compression [38]. Imaging indications have only understated the importance of individualized care with the use of personalized implants such as patient-specific cervical cages or spinal implants customized to fit the patient’s unique anatomy [39]. Tantalum trabecular metal implants in limited studies have illustrated 100% fusion rates after 2 years paired with measures of subsidence decreasing with time, leading to bolstered 2-year clinical outcomes [39].

One of the hallmark indications for surgical intervention remains severe spinal cord compression or where significant narrowing of the spinal canal is evident, commonly secondary to DDD [40]. Provisionally, a successful surgical operation would alleviate the compression and narrowing seen in such patients. Additionally, extensive damage to the vertebral bodies and discs remains a significant indication for cervical corpectomy [21]. In clinical presentation, cervical corpectomy removes the damaged segments, creating space for the spinal cord, relieving pressure, and preventing further structural deterioration. Congenital defects or acquired structural abnormalities by means of trauma or chronic stress resulting in degenerative changes in bone also warrant cervical corpectomy as a viable treatment option [13].

In cases where cervical spine instability or malalignment arises, cervical corpectomy has been cited to improve patient outcomes [41]. By alleviating the symptoms of spinal instability, spinal function is restored, thus improving functional capacity while reducing pain. Regarding neoplastic involvement, cervical corpectomy provides a tangible link in alleviating the orthopedic symptoms associated with the mass but must be reserved for contexts where radiotherapy and chemotherapy’s influence on bone growth is ineffective [42]. A summary of updated clinical indications for cervical corpectomy can be seen below in Table 1.

### 4.2. Surgical Technique

Cervical corpectomies can be performed through various approaches to alleviate neurological defects following degenerative or traumatic disease to the cervical spine. With the advent of novel techniques and technologies, there arises a need to re-evaluate what constitutes the appropriate surgical approach. Specifically, updated indications are necessary, considering the time elapsed since the previous review, as referenced in [22].

**Figure 3:**
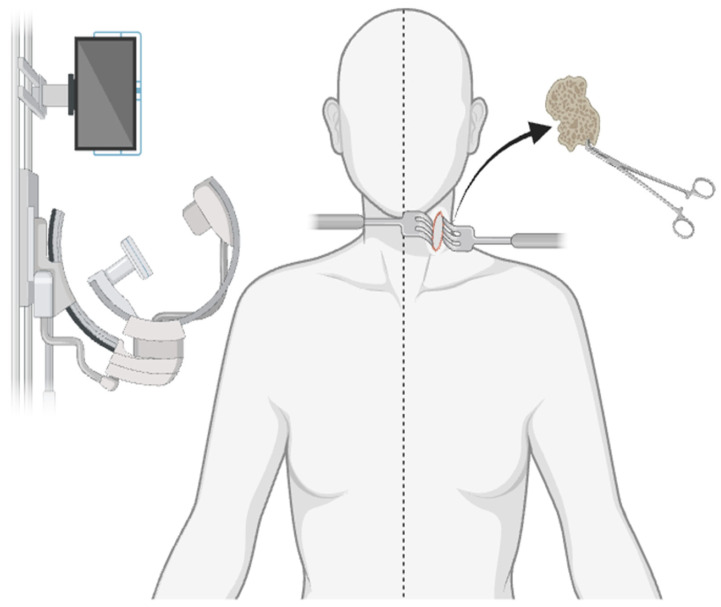
The anterior cervical corpectomy approach. This figure demonstrates several key features of the anterior approach, including neutral neck alignment, lateral fluoroscopy for intraoperative visualization, a 3-inch anterolateral incision, the retraction of soft tissue for a clear surgical field, and ultimately, the removal of pathological bony and/or ligamentous structures.

An anterior cervical corpectomy is the most common cervical corpectomy approach and is performed under general anesthesia with the patient lying on the operating table in the supine position with a neutral neck position [43]. As can be seen in Figure 3, once in position, lateral fluoroscopy is used to assess if the vertebral endplates are positioned parallel to one another, and an AP radiograph is used to assess for neutral axial rotation. An approximately three-inch-long incision is made on the anterolateral aspect of the neck next to the trachea where the muscles of the neck, esophagus, and underlying vasculature are then retracted to expose the cervical spine [44]. Once the cervical spine is exposed, the vertebrae and intervertebral disc of interest are identified. The vertebral body is removed along with any other pathological abnormalities including bone spurs or herniated discs while maintaining the integrity of the neural arch around the spinal cord. Following vertebral removal, the empty space is then filled with either an autograft or allograft. Further, plates, screws, or cages may be employed for support to help the graft fuse with the adjacent vertebrae [45].

Of note, the Minimally Invasive Central Corpectomy (MICC) is a newer procedure in which the removal of a central portion of one or more cervical vertebral bodies and adjacent discs is performed through smaller incisions [46]. With the primary goal of reducing tissue disruption and trauma to surrounding structures, this newer approach enables comparable access to the desired surgical site, while preserving posterior cervical muscle as compared to traditional approaches. Additionally, an endoscopic MICC allows the surgeon to visualize the surgical field using a small camera, thus allowing for a less invasive procedure. Thus, the superiority of the MICC is derived from its soft tissue preserving technique combined with its minimally invasive footprint, allowing for improved postoperative recovery and cervical stability [46]. While an intriguing approach, the MICC is reserved for procedures where the corpectomy retains less than half the vertebral body height to avoid vertebral collapse and kyphotic deformity due to cage subsidence [46]. Moreover, in individuals with significant osteoporosis, a history of heavy smoking, and continual hemodialysis, MICC is strongly contraindicated due to poorer postoperative outcomes.

### 4.3. Patient Outcomes

In the clinical application of spinal decompression and fusion procedures, cervical corpectomies have remained an integral approach that is typically reserved for specific instances and must be evaluated on a case-by-case basis [47]. While remaining a historically safe procedure with low rates of morbidity (1.6% across 1560 procedures) [47], factoring in and accounting for individual anatomy and upper spinal complications is paramount. Recovery is relatively seamless, with some centers reporting a discharge rate of 82.2% within the 3 days following the procedure [48]. Following the results of a systematic review of 240 retrospective studies, prospective studies, and case reports published between 1989 and 2019, an analysis was recovered to illustrate the outcomes and complications associated with cervical corpectomy and anterior cervical spinal surgeries. Particularly, the analysis reveals a plethora of complications, some of which could be potentially life-threatening, while remaining a safe and mainstay procedure. Thus, these findings are crucial for guiding future medical practice and will be further divulged below.

#### 4.3.1. Dysphagia

In terms of adverse events, the most reported postoperative complication was the presence of dysphagia, illustrating a pooled incidence of 5.2% in retrospective studies postoperatively, with only 0.8% of dysphagia remaining as chronic and lasting beyond 3 months [47].

#### 4.3.2. Esophageal Perforation

Esophageal perforation also remains a common complication reported postoperatively in cervical corpectomies, with retrospective studies reporting a pooled incidence of 0.2% (n = 12,842) [47,49,50,51,52].

#### 4.3.3. Laryngeal Nerve Injury

As with any head and neck surgery, the incidence of laryngeal nerve injury remains a possible outcome [53]. Nonetheless, the presence of laryngeal nerve injury was reported in only 1.2% of surgeries (n = 26,464) among retrospective studies [47].

#### 4.3.4. Adjacent Segment Disease

Adjacent segment disease (ASD) is defined as the degenerative process adjacent to a previously fused spine segment and remains a crucial consideration for patients prior to undergoing cervical corpectomy [54]. Drawing from both prospective and retrospective studies, an incidence of 8.6% (n = 2699) of ASD was reported, requiring reoperation [47,55,56,57,58,59,60,61,62,63,64].

#### 4.3.5. Graft and Hardware Failure

In the context of single-level cervical corpectomies, they have been long associated with positive patient outcomes, while multilevel cervical corpectomy—defined as three or more levels—has been linked to drastically worse outcomes [65,66,67]. The analysis of the literature reveals that hardware failure in multilevel cervical corpectomies ranges from 33 to 70%. This figure is in stark contrast to 1–2-level cervical corpectomies, which present in one retrospective longitudinal study as yielding a satisfactory fusion rate in 98.11% of patients [68].

#### 4.3.6. CSF Leak

Cerebrospinal fluid (CSF) leakage is a potential complication when a puncture or perforation allows CSF to escape from the spinal cord, commonly associated with spinal surgery [69]. In terms of cervical corpectomy, a CSF leak is a rare entity with one retrospective study over 10 years illustrating an incidence of 0.7% (n = 1/153) for the treatment of cervical spondylotic myelopathy [70].

#### 4.3.7. Hematoma

A review of 37 retrospective studies within the ranges of 2015–2019 (17 studies), 2010–2014 (11 studies), 2000–2009 (7 studies), and 1989–1999 (2 studies) assessing for postoperative hematoma illustrated an incidence rate of 1.0% with a range of 0–12.5% (n = 865,052). Of studies reporting postoperative hematoma requiring surgical intervention, there was a pooled frequency of 46.1% (n = 1594) [47].

#### 4.3.8. C5 Palsy

Following a review of 19 retrospective studies assessing postoperative C5 palsy, there was a pooled incidence of 3.0% with a range of 0.1–7.7% (n = 5134). Five retrospective studies illustrated non-significant trends towards higher rates of C5 palsy in corpectomy groups [31,71,72].

#### 4.3.9. Infection

Among 46 retrospective studies within the ranges of 2015–2019 (27 studies), 2010–2014 (11 studies), 2000–2009 (4 studies), and 1989–1999 (4 studies), there was a pooled incidence of 1.2%, ranging from 0 to 16% (n = 965,867). Of the 10 studies that specified multiple infection types, bacteremia or sepsis comprised 47.6% of complications related to infection [47,73].

#### 4.3.10. New or Worsened Neurologic Deficits

Finally, with regards to new or worsened neurological deficits following cervical corpectomy, there was an overall incidence of 0.5% across all studies including 15 retrospective studies, 1 prospective study, and 4 case reports. The retrospective study incidence range was 0–25.7% with a pooled incidence of 0.5% (n = 137,654). Some of the new or worsened deficits that occurred following cervical corpectomy included worsened myelopathy due to buckling of the ligamentum flavum, postoperative hemiplegia secondary to spinal cord herniation, postoperative hemiplegia secondary to polymethacrylate extrusion, myelopathy due to intraoperative spinal cord contusion, and transient radiculopathy [47]. A summary of the aforementioned clinical data can be seen below in Table 2.

## 5. Emerging Therapeutic Approaches

### 5.1. Skip Anterior Cervical Corpectomy and Fusion

As described above, one of the largest contributors to adverse outcomes of patients who underwent cervical corpectomy lies within hematomas, adjacent segment disease, and graft and/or hardware failure. Specifically, within the context of multilevel cervical corpectomy, the failure rate of fusion ranges from 33 to 70% across the literature and is the largest hurdle facing modern cervical corpectomy. To address this breakdown in efficacy, clinicians have turned to new surgical approaches, including a novel procedure known as “skip corpectomy”. First described in 2003, skip anterior cervical corpectomy and fusion (sACCF) is defined by corpectomies above and below an intermediate vertebral body, typically the C5 vertebrae, that serves as an additional anchor point to augment biomechanical stability [48]. Specifically, this modality entails a combination of C4 and C6 corpectomy, C5 osteophytectomy, and the dual decompression of the posterior–superior and posterior–inferior aspects of the C5 vertebrae [74]. Further, placement of bone grafts at the C3–C5 and C5–C7 levels is used, in conjunction with the plate fixation of the caudal and rostral vertebrae [74]. In turn, this approach effectively increases bone purchase and strengthens the integrity of the plate construct, in the hopes of decreasing mechanical failure rates following multilevel decompressions.

The most recent study evaluating long-term outcomes related to sACCF included 48 patients who were either diagnosed with cervical spondylotic myelopathy or the ossification of the posterior longitudinal ligament, both common DDD pathologies necessitating multilevel decompression. In Sarigul’s study, fusion was achieved in all patients, and there were no reports of implant-, graft-, or hardware-related complications at the 10th year of the follow-up [75]. A similar study conducted by Monk and colleagues retrospectively analyzed forty-five patients who underwent sACCF, with five patients (11.1%) who developed complications during hospitalization and three (6.7%) who developed instrumentation failure requiring revision at the 10th year of the follow-up [48]. Thus, these mixed results point to sACCF being comparable to ACCF and multilevel ACDF in terms of complication rates and it should be considered in cases where the anterior approach is favorable. Moreover, biomechanical studies reveal that plated skip corpectomies tend to be more stable than standard plated corpectomy, with a reduction in peak screw pull-out force by approximately 15% and 19% in four-screw and six-screw attachment during axial rotation, respectively [76]. Consequently, it is reasonable to infer that skip corpectomy is a safe and effective alternative to standard multilevel corpectomy, especially in higher risk, mobile patients.

### 5.2. Mesh Cage Constructs

Alongside advancements in technique, much research and development has been targeted at materials used in cervical corpectomy. Particularly, an iliac crest autograft, fibular struct graft, and allograft have widely been considered the gold standard graft materials for filling bony defects in corpectomy procedures. However, the morbidity and donor-site complications associated with an autograft, as well as the decreased efficacy with allograft bone, have recently put these materials into question [77]. Hence, the emergence of distractable titanium mesh cages (TMCs) has been developed and biomechanically validated, with studies across the literature purporting significant stability and lower complication rates compared with iliac crest or nondistractable cages [77,78,79]. Through in situ distraction at the site of the defect, the employment of TMCs enables surgeons to restore physiological height, provide immediate strong anterior column support, and avoid bone graft site morbidity. Notably, TMCs are not without disadvantages, particularly concerning cost and the numerous reports of moderate subsidence rates [80].

A second notable material that has recently entered clinical use in corpectomy procedures is biomimetic nanohydroxyapatite/polyamide 66 (n-HA/PA66). In addition to its established use in the integration of autologous bone grafting, as well as transpedicular screw coatings, hydroxyapatite’s (Hap) clinical utility has expanded to cervical corpectomy mesh constructs. Alongside the shared advantage of being an expendable device that allows a better correction of sagittal deformities, non-metallic materials such as n-HA/PA66 are more biocompatible, osteoconductive, and radiolucent as compared to their titanium counterparts [81]. The earliest study comparing the radiographic and clinical outcomes of TMC to n-HA/PA66 in single-level ACCF was conducted by Yang and colleagues in 2013, in which 76 patients were divided into two treatment groups according to device type and were followed for a minimum of four years. This study reported significantly lower rates of subsidence (*p* < 0.0001) in the n-HA/PA66 group compared to TMC, in addition to better radiographic outcomes including segmental sagittal alignment (*p* = 0.235) [82]. Further, the study also reported worse clinical outcomes in the TMC group, as measured by VAS (*p* = 0.034) and JOA (*p* = 0.007) [82]. However, it is important to note that both groups experienced comparable final fusion rates of 97% and 54% for n-HA/PA66 and TMC, respectively [82].

Given the promising results of earlier studies such as that of Yang et al., Li and colleagues set out to evaluate the long-term clinical outcomes of n-HA/PA66 compared to TMC cages in the context of multilevel corpectomy. Using single-institution data from June 2013 to June 2018, the analysis of matched groups similarly yielded significantly lower rates of subsidence with the use of n-HA/PA66 compared to TMC (12% vs. 40%, *p* = 0.024) [83]. By extension, Li et al. also reported significantly lower rates of postoperative complications including adjacent segment disease, with an occurrence of 16% in the n-HA/PA66 group compared to 44% in the TMC group (*p* = 0.031) [83]. However, there was not a statistically significant difference in reported patient outcomes as measured by VAS and JOA at any time between the two groups. Given the results of these studies and the like, the n-HA/PA66 cage is associated with excellent radiographic fusion, lower subsidence, and equal-to-slightly-better clinical outcomes than TMC in both the short term and long term after cervical corpectomy. Thus, with the added benefit of radiolucency, the employment of n-HA/PA66 over TMC is both a safe and effective treatment option for future practitioners.

## 6. Conclusions

This review highlights the recent advancement in cervical corpectomy as a surgical intervention for cervical DDD and other related spinal conditions. The emergence of new technologies in imaging and arthroplasty implant devices alongside advancements in new surgical approaches has significantly contributed to more precise and effective treatment of cervical spine conditions. Particularly, this review highlighted clinical indications for cervical corpectomy, including severe spinal cord or nerve root compression, significant vertebral body and disc damage, and other structural abnormalities. The progression of surgical techniques, from the standard anterior cervical corpectomy to more minimally invasive procedures like endoscopic MICC, reflects on the growth of surgical advancement to improve patient outcomes and minimize postoperative complications. While many patients experience significant symptom relief and improved quality of life postoperatively, factors such as the patient’s overall health, the severity and nature of their spinal condition, and the specific surgical approach employed all play a role in the variability seen in postoperative outcomes following cervical corpectomy. Thus, this emphasizes the need for a personalized, multidisciplinary approach in patient management, involving not only surgeons but also physiotherapists, pain specialists, and other healthcare professionals to optimize patient recovery and rehabilitation. Furthermore, this review underscores that despite the reports of good clinical outcomes, cervical corpectomy is not without risks. The incidence of complications such as dysphagia, esophageal perforation, laryngeal nerve injury, adjacent segment disease, graft and hardware failure, and cerebrospinal fluid leaks, while relatively low, emphasizes the need for continued focus on preoperative planning, refining surgical techniques, and the development of novel therapeutics and devices. Consequently, this review concludes with a discussion of new techniques such as sACCF and the advent of integrating superiorly biocompatible materials such as n-HA/PA66 into distractable mesh cages for single- and multilevel cervical corpectomy for improved functional outcomes and blunted complication rates.

## Figures and Tables

**Table 1:** Clinical indications and criteria for cervical corpectomy.

Clinical Indications and Criteria for Cervical Corpectomy	
Indication for Cervical Corpectomy	Criteria
1. Severe Spinal Cord or Nerve Root Compression	Radiological evidence of severe spinal cord compression Presence of myelopathy with neurological deficits Demonstrated spinal cord or nerve root compression on CT myelogram or imaging studies
2. Vertebral Body and Disc Damage	Significant damage to the vertebral bodies and adjacent intervertebral discs Instability or collapse of vertebral segments
3. Structural Abnormalities	Congenital or acquired structural abnormalities, such as severe kyphosis or scoliosis Presence of deformities
4. Cervical Spine Instability or Malalignment	Evidence of cervical spine instability or malalignment on imaging studies
5. Tumor or Neoplastic Involvement	Presence of cervical spine tumors or neoplastic lesions requiring surgical excision
6. Failed Conservative Treatment	Inadequate response to non-surgical treatments, such as physical therapy, medications, or immobilization

**Table 2:** Complications and outcomes in cervical corpectomy.

Complications and Outcomes in Cervical Corpectomy	
Complication Category	Description and Incidence (%)
Dysphagia and Swallowing Difficulties	Transient dysphagia = 5.2% (n = 737,041) Persistent dysphagia = 0.8% (n = 2122)
Esophageal Perforation	Incidence of esophageal perforation = 0.2% (n = 12,842)
Laryngeal Nerve Injury	Recurrent Laryngeal Nerve Palsy = 1.2% (n = 26,464)
Adjacent Segment Disease	ASD Requiring Reoperation = 8.6% (n = 2699)
Graft and Hardware Failure	1–2-Level Cervical Corpectomy = 98.11% success rate (n = 52)
CSF Leak	Postoperative CSF leak incidence = 0.7% (n = 1)
Hematoma	Incidence of postoperative hematoma = 1.0% (n = 865,052) Incidence of postoperative hematoma requiring surgical intervention = 46.1% (n = 1594)
C5 Palsy	Incidence of postoperative C5 palsy = 3.0% (n = 5134)
Infection	Incidence of postoperative infection = 1.2% (n = 965,867)
New or Worsened Neurological Deficit	Incidence of deficit = 0.5% (n = 137,654)

## References

[1] Özgen S., Naderi S., Özek M.M., Pamir M.N. (2004). A retrospective review of cervical corpectomy: Indications, complications and outcome. Acta Neurochir..

[2] Foreman M., Patel A., Nguyen A., Foster D., Orriols A., Lucke-Wold B. (2024). Management Considerations for Total Intervertebral Disc Replacement. World Neurosurg..

[3] Fehlings M.G., Arvin B. (2009). Surgical management of cervical degenerative disease: The evidence related to indications, impact, and outcome. J. Neurosurg. Spine.

[4] Hammer C., Heller J., Kepler C. (2016). Epidemiology and pathophysiology of cervical disc herniation. Semin. Spine Surg..

[5] Fakhoury J., Dowling T.J. (2023). Cervical Degenerative Disc Disease. StatPearls.

[6] Choi Y.S. (2009). Pathophysiology of Degenerative Disc Disease. Asian Spine J..

[7] de Castro I., dos Santos D.P., de Christoph D.H., Landeiro J.A. (2005). The history of spinal surgery for disc disease: An illustrated timeline. Arq. Neuropsiquiatr..

[8] Denaro V., Di Martino A. (2011). Cervical spine surgery: An historical perspective. Clin. Orthop. Relat. Res..

[9] Hutchings M.I., Truman A.W., Wilkinson B. (2019). Antibiotics: Past, present and future. Curr. Opin. Microbiol..

[10] Smith G.W., Robinson R.A. (1958). The treatment of certain cervical-spine disorders by anterior removal of the intervertebral disc and interbody fusion. J. Bone Jt. Surg. Am..

[11] (2005). Extensile exposure. By Arnold K. Henry. Emeritus Professor of Clinical Surgery, University of Egypt; and Professor of Anatomy, Royal College of Surgeons, Ireland. Second edition. 64 × 92 in. Pp. 320, with 298 illustrations. 1957. Edinburgh: E. & S. Livingstone Ltd. 45s. Br. J. Surg..

[12] Dweik A., Van Den Brande E., Kossmann T., Maas A.I.R. (2013). History of cervical spine surgery: From nihilism to advanced reconstructive surgery. Spinal Cord..

[13] Tatter C., Persson O., Burström G., Edström E., Elmi-Terander A. (2020). Anterior Cervical Corpectomy and Fusion for Degenerative and Traumatic Spine Disorders, Single-Center Experience of a Case Series of 119 Patients. Oper. Neurosurg..

[14] Robinson R.A., Smith G.W. (2010). Anterolateral cervical disc removal and interbody fusion for cervical disc syndrome. Sas J..

[15] Virk S., Qureshi S., Sandhu H. (2020). History of Spinal Fusion: Where We Came from and Where We Are Going. HSS J..

[16] Aronson N., Filtzer D.L., Bagan M. (1968). Anterior cervical fusion by the smith-robinson approach. J. Neurosurg..

[17] Vigo V., Pastor-Escartín F., Doniz-Gonzalez A., Quilis-Quesada V., Capilla-Guasch P., González-Darder J.M., De Bonis P., Fernandez-Miranda J.C. (2020). The Smith-Robinson Approach to the Subaxial Cervical Spine: A Stepwise Microsurgical Technique Using Volumetric Models From Anatomic Dissections. Oper. Neurosurg..

[18] Boni M., Cherubino P., Denaro V., Benazzo F. (1984). Multiple subtotal somatectomy. Technique and evaluation of a series of 39 cases. Spine.

[19] Peterson J.C., Arnold P.M., Smith Z.A., Hsu W.K., Fehlings M.G., Hart R.A., Hilibrand A.S., Nassr A., Rahman R.K., Tannoury C.A. (2017). Misplaced Cervical Screws Requiring Reoperation. Glob. Spine J..

[20] Fineberg S.J., Ahmadinia K., Oglesby M., Patel A.A., Singh K. (2013). Hospital outcomes and complications of anterior and posterior cervical fusion with bone morphogenetic protein. Spine.

[21] Williams K.E., Paul R., Dewan Y. (2009). Functional outcome of corpectomy in cervical spondylotic myelopathy. Indian J. Orthop..

[22] Medow J.E., Trost G., Sandin J. (2006). Surgical management of cervical myelopathy: Indications and techniques for surgical corpectomy. Spine J..

[23] Cummins B.H., Robertson J.T., Gill S.S. (1998). Surgical experience with an implanted artificial cervical joint. J. Neurosurg..

[24] Singh K., Vaccaro A.R., Kim J., Lorenz E.P., Lim T.H., An H.S. (2004). Enhancement of Stability Following Anterior Cervical Corpectomy: A Biomechanical Study. Spine.

[25] Mao N., Wu J., Zhang Y., Gu X., Wu Y., Lu C., Ding M., Lv R., Li M., Shi Z. (2015). A Comparison of Anterior Cervical Corpectomy and Fusion Combined With Artificial Disc Replacement and Cage Fusion in Patients With Multilevel Cervical Spondylotic Myelopathy. Spine.

[26] Eleraky M.A., Llanos C., Sonntag V.K.H. (1999). Cervical corpectomy: Report of 185 cases and review of the literature. J. Neurosurg. Spine.

[27] Xiao S.W., Jiang H., Yang L.J., Xiao Z.M. (2015). Anterior cervical discectomy versus corpectomy for multilevel cervical spondylotic myelopathy: A meta-analysis. Eur. Spine J..

[28] Oh M.C., Zhang H.Y., Park J.Y., Kim K.S. (2009). Two-Level Anterior Cervical Discectomy Versus One-Level Corpectomy in Cervical Spondylotic Myelopathy. Spine.

[29] Wang T., Wang H., Liu S., An H.D., Liu H., Ding W.Y. (2016). Anterior cervical discectomy and fusion versus anterior cervical corpectomy and fusion in multilevel cervical spondylotic myelopathy. Medicine.

[30] Jiang S.D., Jiang L.S., Dai L.Y. (2012). Anterior cervical discectomy and fusion versus anterior cervical corpectomy and fusion for multilevel cervical spondylosis: A systematic review. Arch. Orthop. Trauma Surg..

[31] Xu H.-D., Du J.-Y., Ling Z.-H., Ling X.-J., Wen Z.-Q. (2015). Anterior cervical discectomy and fusion versus anterior cervical corpectomy and fusion in the treatment of multilevel cervical spondylotic myelopathy: Systematic review and a meta-analysis. Ther. Clin. Risk Manag..

[32] Louie P.K., Nemani V.M., Leveque J.C.A. (2022). Anterior Cervical Corpectomy and Fusion for Degenerative Cervical Spondylotic Myelopathy: Case Presentation With Surgical Technique Demonstration and Review of Literature. Clin. Spine Surg..

[33] Parker S.L., Wolinsky J.P., Tufaro A.P., Gokaslan Z.L., Witham T.F. (2015). Five-level cervical corpectomy for neurofibromatosis-associated spinal deformity: Case report. Eur. Spine J..

[34] Hartmann S., Tschugg A., Obernauer J., Neururer S., Petr O., Thomé C. (2016). Cervical corpectomies: Results of a survey and review of the literature on diagnosis, indications, and surgical technique. Acta Neurochir..

[35] Perez-Cruet M.J., Samartzis D., Fessler R.G. (2006). Anterior Cervical Discectomy and Corpectomy. Oper. Neurosurg..

[36] Bailey R.W., Badgley C.E. (1960). Stabilization of the cervical spine by anterior fusion. J. Bone Jt. Surg. Am..

[37] Cloward R.B. (1958). The anterior approach for removal of ruptured cervical disks. J. Neurosurg..

[38] Desai A., Pendharkar A.V., Swienckowski J.G., Ball P.A., Lollis S., Simmons N.E. (2015). Utility of Routine Outpatient Cervical Spine Imaging Following Anterior Cervical Corpectomy and Fusion. Cureus.

[39] King V., Swart A., Winder M.J. (2016). Tantalum trabecular metal implants in anterior cervical corpectomy and fusion: 2-year prospective analysis. J. Clin. Neurosci..

[40] Pescatori L., Tropeano M.P., Visocchi M., Grasso G., Ciappetta P. (2020). Cervical Spondylotic Myelopathy: When and Why the Cervical Corpectomy?. World Neurosurg..

[41] Elsissy J., Kutzner A., Danisa O. (2019). Delayed Diagnosis and Management of Traumatic Cervical Spine Subluxation. J. Orthop. Case Rep..

[42] Eleraky M., Setzer M., Vrionis F.D. (2010). Posterior transpedicular corpectomy for malignant cervical spine tumors. Eur. Spine J..

[43] Leven D., Meaike J., Radcliff K., Qureshi S. (2017). Cervical disc replacement surgery: Indications, technique, and technical pearls. Curr. Rev. Musculoskelet Med..

[44] Peloza J., Malone H., Jacobian E., Kolsky D.E., Harel R., Guyer R.D., Millgram M.A., Ashkenazi E. (2023). The use of a new high-speed shielded curved drill is associated with improved intraoperative and clinical outcomes after cervical corpectomy and fusion procedures: A retrospective case series. J. Orthop. Surg. Res..

[45] Tohamy M.H., Osterhoff G., Abdelgawaad A.S., Ezzati A., Heyde C.E. (2022). Anterior cervical corpectomy and fusion with stand-alone cages in patients with multilevel degenerative cervical spine disease is safe. BMC Musculoskelet Disord..

[46] Hirano Y., Mizuno J., Nakagawa H., Itoh Y., Kubota K., Watanabe S., Matsuoka H., Numazawa S., Tomii M., Watanabe K. (2011). Minimally invasive central corpectomy for ossified posterior longitudinal ligament in the cervical spine. J. Clin. Neurosci..

[47] Yee T.J., Swong K., Park P. (2020). Complications of anterior cervical spine surgery: A systematic review of the literature. J. Spine Surg..

[48] Monk S.H., O’brien M., Perle S., Bohl M., Finger F., Chewning S.J., Holland C.M. (2023). Ten-Year Experience of Skip Anterior Cervical Corpectomy and Fusion. Int. J. Spine Surg..

[49] Zhong Z.-M., Jiang J.-M., Qu D.-B., Wang J., Li X.-P., Lu K.-W., Xu B., Chen J.-T. (2013). Esophageal perforation related to anterior cervical spinal surgery. J. Clin. Neurosci..

[50] Lee D.-H., Cho J.H., Hwang C.J., Lee C.S., Cho S.K., Kim C., Ha J.-K. (2018). What Is the Fate of Pseudarthrosis Detected 1 Year After Anterior Cervical Discectomy and Fusion?. Spine.

[51] Huang J.J., Niu C.C., Chen L.H., Lai P.L., Fu T.S., Chen W.J. (2004). Anterior cervical spinal surgery for multilevel cervical myelopathy. Chang. Gung Med. J..

[52] Yagi K., Nakagawa H., Okazaki T., Irie S., Inagaki T., Saito O., Nagahiro S., Saito K. (2017). Noninfectious prevertebral soft-tissue inflammation and hematoma eliciting swelling after anterior cervical discectomy and fusion. J. Neurosurg. Spine.

[53] Culp J.M., Patel G. (2024). Recurrent Laryngeal Nerve Injury. StatPearls.

[54] McDonald C.L., Alsoof D., Glueck J., Osorio C., Stone B., McCluskey L., Diebo B.G., Daniels A.H., Basques B.A. (2023). Adjacent Segment Disease After Spinal Fusion. JBJS Rev..

[55] Skeppholm M., Lindgren L., Henriques T., Vavruch L., Löfgren H., Olerud C. (2015). The Discover artificial disc replacement versus fusion in cervical radiculopathy--a randomized controlled outcome trial with 2-year follow-up. Spine J..

[56] Gok B., Sciubba D.M., McLoughlin G.S., McGirt M., Ayhan S., Wolinsky J.-P., Bydon A., Gokaslan Z.L., Witham T.F. (2008). Surgical treatment of cervical spondylotic myelopathy with anterior compression: A review of 67 cases. J. Neurosurg. Spine.

[57] Papadopoulos E.C., Huang R.C., Girardi F.P., Synnott K., Cammisa F.P. (2006). Three-level anterior cervical discectomy and fusion with plate fixation: Radiographic and clinical results. Spine.

[58] Song K.J., Taghavi C.E., Lee K.B., Song J.H., Eun J.P. (2009). The efficacy of plate construct augmentation versus cage alone in anterior cervical fusion. Spine.

[59] De la Garza-Ramos R., Xu R., Ramhmdani S., Kosztowski T., Bydon M., Sciubba D.M., Wolinsky J.-P., Witham T.F., Gokaslan Z.L., Bydon A. (2016). Long-term clinical outcomes following 3- and 4-level anterior cervical discectomy and fusion. J. Neurosurg. Spine.

[60] Scholz T., Mainz V., Blume C., Albanna W., Clusmann H., Müller A., Geiger M.F. (2018). Anterior Cervical Decompression and Fusion or Posterior Foraminotomy for Cervical Radiculopathy: Results of a Single-Center Series. J. Neurol. Surg. A Cent Eur. Neurosurg..

[61] Lonjon N., Favreul E., Huppert J., Lioret E., Delhaye M., Mraidi R. (2019). Clinical and radiological outcomes of a cervical cage with integrated fixation. Medicine.

[62] Kaiser M.G., Haid R.W., Subach B.R., Barnes B., Rodts G.E. (2002). Anterior cervical plating enhances arthrodesis after discectomy and fusion with cortical allograft. Neurosurgery.

[63] Alhashash M., Shousha M., Boehm H. (2018). Adjacent Segment Disease After Cervical Spine Fusion: Evaluation of a 70 Patient Long-Term Follow-Up. Spine.

[64] Narain A.S., Hijji F.Y., Haws B.E., Kudaravalli K.T., Yom K.H., Markowitz J., Singh K. (2018). Impact of body mass index on surgical outcomes, narcotics consumption, and hospital costs following anterior cervical discectomy and fusion. J. Neurosurg. Spine.

[65] Vaccaro A.R., Falatyn S.P., Scuderi G.J., Eismont F.J., McGuire R.A., Singh K., Garfin S.R. (1998). Early failure of long segment anterior cervical plate fixation. J. Spinal Disord..

[66] Wang J.C., Hart R.A., Emery S.E., Bohlman H.H. (2003). Graft migration or displacement after multilevel cervical corpectomy and strut grafting. Spine.

[67] Macdonald R.L., Fehlings M.G., Tator C.H., Lozano A., Fleming J.R., Gentili F., Bernstein M., Wallace M.C., Tasker R.R. (1997). Multilevel anterior cervical corpectomy and fibular allograft fusion for cervical myelopathy. J. Neurosurg..

[68] Tome-Bermejo F., Alvarez-Galovich L., Pinera-Parrilla A.R., Mengis-Palleck C.L., Cervera-Irimia J., Rodriguez-Bercial A., Moreno-Mateo F., Sutil-Blanco A. (2022). Anterior 1-2 Level Cervical Corpectomy and Fusion for Degenerative Cervical Disease: A Retrospective Study With Lordotic Porous Tantalum Cages. Long-Term Changes in Sagittal Alignment and Their Clinical and Radiological Implications After Cage Subsidence. Int. J. Spine Surg..

[69] Fang Z., Tian R., Jia Y.T., Xu T.T., Liu Y. (2017). Treatment of cerebrospinal fluid leak after spine surgery. Chin. J. Traumatol..

[70] Chacko A.G., Turel M.K., Sarkar S., Prabhu K., Daniel R.T. (2014). Clinical and radiological outcomes in 153 patients undergoing oblique corpectomy for cervical spondylotic myelopathy. Br. J. Neurosurg..

[71] Lin Q., Zhou X., Wang X., Cao P., Tsai N., Yuan W. (2012). A comparison of anterior cervical discectomy and corpectomy in patients with multilevel cervical spondylotic myelopathy. Eur. Spine J..

[72] Lau D., Chou D., Mummaneni P.V. (2015). Two-level corpectomy versus three-level discectomy for cervical spondylotic myelopathy: A comparison of perioperative, radiographic, and clinical outcomes. J. Neurosurg. Spine.

[73] Segal D.N., Wilson J.M., Staley C., Yoon S.T. (2019). Outpatient and Inpatient Single-level Cervical Total Disc Replacement: A Comparison of 30-day Outcomes. Spine.

[74] Dalbayrak S., Yilmaz M., Naderi S. (2010). “Skip” corpectomy in the treatment of multilevel cervical spondylotic myelopathy and ossified posterior longitudinal ligament. J. Neurosurg. Spine.

[75] Sarigul B., Ogrenci A., Yilmaz M., Dalbayrak S. (2023). “Skip” corpectomy technique in multilevel cervical spondylotic myelopathy and ossified posterior longitudinal ligament: Outcomes with over 10-years follow-up. Br. J. Neurosurg..

[76] Yilmaz M., Yüksel K.Z., Baek S., Newcomb A.G., Dalbayrak S., Sonntag V.K.M., Crawford N.R. (2017). Biomechanics of Cervical “Skip” Corpectomy Versus Standard Multilevel Corpectomy. Clin. Spine Surg..

[77] Burkett C.J., Baaj A.A., Dakwar E., Uribe J.S. (2012). Use of titanium expandable vertebral cages in cervical corpectomy. J. Clin. Neurosci..

[78] Woiciechowsky C. (2005). Distractable Vertebral Cages for Reconstruction After Cervical Corpectomy. Spine.

[79] Dorai Z., Morgan H., Coimbra C. (2003). Titanium cage reconstruction after cervical corpectomy. J. Neurosurg. Spine.

[80] Ji C., Yu S., Yan N., Wang J., Hou F., Hou T., Cai W. (2020). Risk factors for subsidence of titanium mesh cage following single-level anterior cervical corpectomy and fusion. BMC Musculoskelet. Disord..

[81] Papanastassiou I.D., Gerochristou M., Aghayev K., Vrionis F.D. (2013). Defining the indications, types and biomaterials of corpectomy cages in the thoracolumbar spine. Expert Rev. Med. Devices.

[82] Yang X., Chen Q., Liu L., Song Y., Kong Q., Zeng J., Xue Y., Ren C. (2013). Comparison of anterior cervical fusion by titanium mesh cage versus nano-hydroxyapatite/polyamide cage following single-level corpectomy. Int. Orthop. (SICOT).

[83] Li Q., Hu B., Masood U., Zhang Z., Yang X., Liu L., Feng G., Yang H., Song Y. (2023). A Comparison of Corpectomy ACDF Hybrid Procedures with Nano-Hydroxyapatite/Polyamide 66 Cage and Titanium Mesh Cage for Multi-level Degenerative Cervical Myelopathy: A Stepwise Propensity Score Matching Analysis. Orthop. Surg..

